# Moderate Ohmic Field Modification of Okara and Its Effects on Physicochemical Properties, Structural Organization, and Functional Characteristics

**DOI:** 10.3390/foods14101833

**Published:** 2025-05-21

**Authors:** Zhongwen Cao, Chengcheng Xie, Cheng Yang, Xingyu Liu, Xiangren Meng

**Affiliations:** 1 School of Tourism and Cuisine, Yangzhou University, Yangzhou 225127, China; 19895325091@163.com (C.X.); 17851974918@163.com (X.L.); 2 Key Laboratory of Chinese Cuisine Intangible Cultural Heritage Technology Inheritance, Ministry of Culture and Tourism, Yangzhou 225127, China; 19551651340@163.com; 3 School of Food Science and Engineering, Yangzhou University, Yangzhou 225127, China

**Keywords:** ohmic heating, okara, modification, functional foods

## Abstract

This study employed ohmic heating to investigate its impact on the physicochemical properties, structural organization, and functional characteristics of okara. Ohmic heating was applied with different field strengths and holding times. After moderate ohmic treatment, the water-holding capacity, oil-holding capacity, and swelling capacity of okara increased by 51.11%, 88.89%, and 43.64%, respectively. The microstructure and secondary structure were improved. The total sugar and soluble dietary fiber content were enhanced. The levels of active substances such as total flavonoids and total phenols significantly increased, leading to improved antioxidant capacity. The properties of okara were influenced by the field strength and holding time. This study provides new insights for the processing and development of okara, particularly in the application of functional foods.

## 1. Introduction

Okara is a by-product generated during the production of soy milk or tofu, with approximately 1.2 kg of fresh okara produced for every 1 kg of tofu processed. Globally, around 140 million tonnes of okara are produced annually [1]. However, it is frequently discarded without pretreatment, leading to environmental pollution, or repurposed as animal feed or fertilizer, failing to realize its full economic potential. Okara is composed of approximately 50% dietary fiber, 25% protein, 10% fat, 5% carbohydrates, and 5% ash [2]. It can serve as a source to complement the limiting amino acids in cereal products, especially lysine [3]. The consumption of okara is beneficial to human health, as it can regulate blood sugar levels, reduce blood lipids, and help prevent obesity and cancer [4].

Currently, the primary methods of okara modification encompass physical, chemical, biological, and combined approaches, which have notable effects on the structural functionality and biological properties of okara. Physical methods primarily involve high hydrostatic pressure [5], homogenization [6], steam explosion [7], nanocellulose technology [8], extrusion [9], microwave treatment [10], and ultrasound treatment [11], among other techniques. Chemical methods commonly include alkali extraction and acid precipitation [12,13]. Biological methods entail the utilization of microbial fermentation for okara modification [14,15]. Furthermore, there are combined modification technologies. For instance, Lin et al. [16] investigated the conversion of insoluble dietary fiber (IDF) in okara to soluble dietary fiber (SDF) through a method that integrates high-pressure homogenization with microbial fermentation. Research by Lei et al. [17] indicates that post-microbial fermentation UV-A irradiation is beneficial for improving the physicochemical properties and functionality of okara. Integrating dry planetary ball milling with fermentation is advantageous for enhancing the prospects of okara’s application in functional foods [18]. Ohmic heating (OH) is a novel heating method widely utilized for its rapid and uniform heat generation with high energy efficiency. In previous studies, OH has been predominantly utilized in food processing applications such as cooking [19], thawing [20], blanching [21], and sterilization [22], and has consistently received favorable evaluations. In recent years, its application has expanded to the extraction and modification of bioactive compounds and nutrients. Compared with conventional thermal processing methods, OH has been reported to exert less detrimental effects on bioactive compounds [23], while also offering greater environmental sustainability, higher extraction efficiency, and reduced processing time [24]. Specifically, OH extraction has been shown to reduce energy consumption by 63% and to yield higher quantities of bioactive substances with enhanced antioxidant properties [25]. Additionally, studies have demonstrated that OH facilitates the extraction of natural pigments from red beetroot [26]. In the context of modification, OH has been found to improve the structural and flavor characteristics of peanut protein [27]. The application of OH in the processing of by-products has also attracted considerable attention [28]. OH treatment has been shown to enhance the extraction of bioactive phenolic compounds from cocoa bean husks [29]. Similarly, it significantly increased the concentration of total phenolic compounds in grape skin extracts [30]. Furthermore, OH modification improved the bioactivity and nutritional value of sugarcane bagasse [31].

The aim of this study was to employ OH for the modification of okara and to investigate its effects on the physical properties, microstructure, and bioactive components of okara. The objective was to enhance the functional characteristics of okara, minimize resource waste, and offer new perspectives for its application in food products, particularly in the development of functional foods.

## 2. Materials and Methods

### 2.1. Materials

The okara was obtained from Qilu Flavor (Linyi, China), dried in a 60 °C oven for 6 h, ground into powder, and sieved through an 80-mesh screen for particle size uniformity.

### 2.2. Sample Preparation

#### 2.2.1. OH Treatment

**Figure 1:**
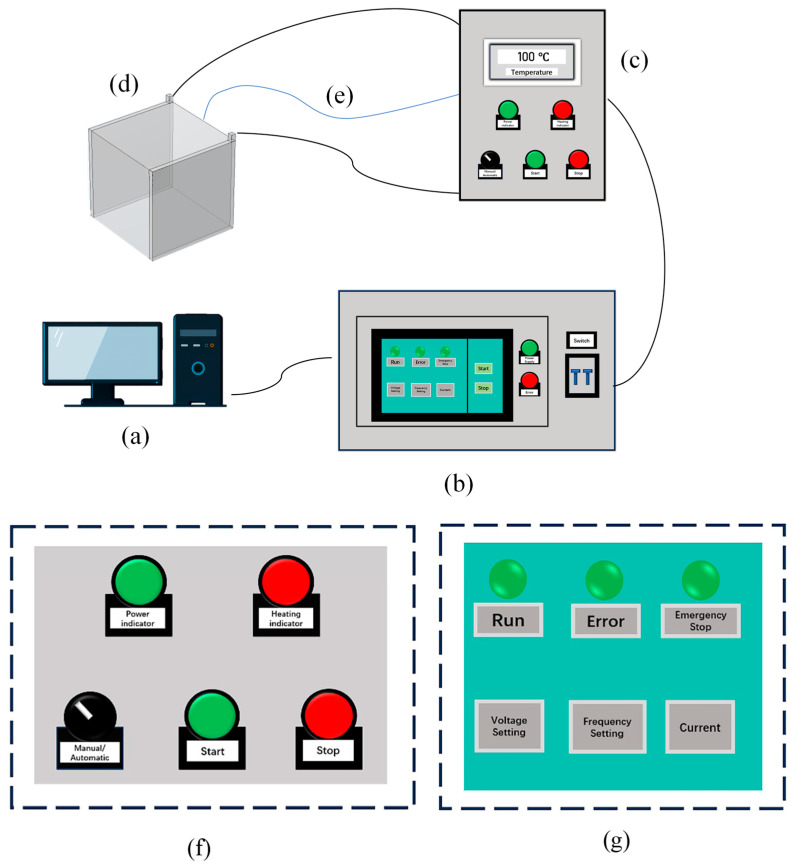
OH Schematic Diagram: (**a**) control computer, (**b**) alternating current variable-frequency power supply, (**c**) control cabinet, (**d**) heating chamber, (**e**) thermocouples, (**f**) control panel of alternating current variable-frequency power supply, and (**g**) control panel of control cabinet. OH: Ohmic heating.

The OH treatment was performed using a laboratory-fabricated ohmic heating system. As illustrated in Figure 1, the apparatus comprised an AC variable-frequency power supply (ANJ13-1kVA, Anais Power Supply, Suzhou, China), a needle-type thermocouple (HY005, Fuyang Instrumentation, Guangzhou, China), a control computer, a quartz heating chamber (internal dimensions: 7.6 × 7.6 × 7.8 cm), and two parallel stainless steel electrodes (7.6 × 0.2 × 7.8 cm) positioned within the chamber. Okara was mixed with deionized water at a solid-to-liquid ratio of 1:10 and introduced into the heating chamber. The chamber was placed on a magnetic stirrer to ensure sample homogeneity. During the heating process, alternating current electric field strengths of 40, 45, and 50 V/cm were applied at a frequency of 2000 Hz. The samples were heated to 100 °C and then held at this temperature for 3, 6, and 9 min, corresponding to groups labeled as OH40-3, OH40-6, OH40-9, OH45-3, OH45-6, OH45-9, OH50-3, OH50-6, and OH50-9. Subsequently, the samples were collected, freeze-dried, and sieved through an 80-mesh screen for further use.

#### 2.2.2. Blank Group and Control Group

The blank group consisted of untreated okara, labeled as UT. The control group utilized water bath heating (WH), where samples with a solid–liquid ratio of 1:10 were heated in a water bath at 25 °C until reaching 100 °C, followed by incubation for 3, 6, and 9 min before removal (designated as WH0-3, WH0-6, and WH0-9). Subsequently, the samples were freeze-dried, sieved through an 80-mesh screen, and kept for further use.

### 2.3. Water-Holding Capacity and Oil-Holding Capacity (WHC and OHC)

The okara was mixed with water or soybean oil in a 1:10 (*w*/*v*) ratio and vortexed for 1 min to ensure thorough mixing. Subsequently, the mixture was centrifuged at 8000× *g* for 10 min. The supernatant was discarded, and the test tubes containing the sediment were weighed. The water-holding capacity (WHC) or oil-holding capacity (OHC) was calculated as the grams of water or oil absorbed and retained per gram of sample [32].

WHC or OHC (g/g) = (m2 − m1)/m1(1)

where

m1 is the weight of the sample, in g;
m2 is the weight of the test tube with sediment, in g.

### 2.4. Swelling Capacity (SC) Analysis

A sample weighing 1.00 g, denoted as m0, was taken. In a 10 mL graduated cylinder, ensuring the sample surface was level, the initial volume, V1, was accurately read and recorded. Distilled water was then added in a 1:10 ratio, and the mixture was stirred until homogeneous. After allowing the sample to rest undisturbed on a level surface for 24 h, the final volume after complete swelling, V2, was accurately recorded [33].

SC (mL/g) = (V2 − V1)/m0(2)

### 2.5. Total Sugar Content Analysis

The determination of total sugar content was conducted with reference to Song et al. [34] with slight modifications. Total sugar content was calculated using the phenol–sulfuric acid method. Standard curves were prepared using different concentrations of glucose solutions, and the absorbance was measured at 540 nm.

### 2.6. Analysis of Bioactive Compounds

#### 2.6.1. Soluble Dietary Fiber (SDF)

The SDF content of modified okara was determined using the AOAC method [35].

#### 2.6.2. Total Flavonoid Content (TFC)

The total flavonoid content (TFC) analysis of okara followed a method slightly modified from Song et al. [36]. In this method, 500 μL of the extract (1 mg/mL in distilled water) was added to 1 mL of NaNO_2_ (5%) and thoroughly mixed. After 6 min, 1 mL of 10% AlCl_3_ was added to the mixture, followed by the addition of 10 mL of NaOH (1 M), and the volume of the mixture was adjusted to 25 mL with distilled water. The reaction mixture was then allowed to stand at room temperature for 15 min, and the absorbance at 510 nm was recorded. A calibration curve was plotted using rutin standard solutions (0–250 μg/mL) [37].

#### 2.6.3. Total Phenolic Content (TPC)

The total phenolic content (TPC) of okara extract was determined following the method described by Song et al. [36] using the Folin–Ciocalteu reagent. In this method, 1 mL of the extract (1 mg/mL in distilled water) was added to 1 mL of Folin–Ciocalteu reagent. After 3 min, 1 mL of saturated Na_2_CO_3_ (35%) was added to the mixture. The volume of the mixture was then adjusted to 10 mL with distilled water. The reaction mixture was then kept in the dark for 90 min, and the absorbance at 765 nm was recorded. A calibration curve was plotted using gallic acid standard solutions (0–250 μg/mL) [37].

### 2.7. Antioxidant Activity

The supernatant used for antioxidant capacity determination was the same as the supernatant used for measuring TPC and TFC, both of which are okara extracts (1 mg/mL in distilled water).

#### 2.7.1. ABTS

The ABTS assay consisted of a sample group (As), a control group (Ab), and a blank group (Ac). In total, 3.9 mL of ABTS solution (7 mmol/L ABTS and 2.45 mmol/L potassium persulfate) was mixed with 0.1 mL of the supernatant for the sample group; 0.1 mL of deionized water was mixed with 3.9 mL of ABTS solution for the blank group; and 0.1 mL of sample supernatant was mixed with 3.9 mL of deionized water for the control group. The mixtures were shaken well and allowed to stand for 6 min in a dark room at room temperature before measuring the absorbance at 734 nm [38].

#### 2.7.2. DPPH

The DPPH assay consisted of a sample group (As), a control group (Ab), and a blank group (Ac). In total, 2 mL of supernatant was placed into a test tube, followed by the addition of 2 mL of DPPH ethanol solution (71 µmol/L) for the sample group; 2 mL of supernatant was placed into a test tube, followed by the addition of 2 mL of anhydrous ethanol solution for the blank group; and 2 mL of anhydrous ethanol solution was placed into a test tube, followed by the addition of 2 mL of DPPH ethanol solution for the control group. The mixtures were shaken well and allowed to stand for 30 min in a dark room at room temperature before measuring the absorbance at 517 nm [39].

### 2.8. Fourier-Transform Infrared Spectroscopy (FTIR)

A total of 1 mg of freeze-dried sample was ground and mixed with 100 mg of potassium bromide in an agate mortar, and pressed into a thin pellet. The sample was analyzed using a Fourier-transform infrared spectrometer (Frontier, PerkinElmer, Boston, MA, USA) with a scanning range from 4000 to 450 cm^−1^ [40].

### 2.9. Electron Microscopy

The surface microstructure of the okara sample was observed using a scanning electron microscope (S-3400 N, SEM, Hitachi Ltd., Tokyo, Japan). Prior to analysis, the sample underwent freeze-drying, grinding, sieving, and ion sputter coating with gold [41].

### 2.10. Data Analysis

Each experiment was repeated at least three times. The data were analyzed using IBM SPSS Statistics 27, and the results were presented as mean ± standard deviation (SD). Analysis of variance (ANOVA) was conducted to test for significant differences, followed by Duncan’s multiple range test with a significance level of *p* < 0.05. Graphs were plotted using Origin 2021.

## 3. Results and Discussion

### 3.1. WHC and OHC

WHC refers to the ability of okara to retain moisture when subjected to external forces [42], while OHC denotes its capability to absorb fats. These properties play a significant role in inhibiting cholesterol or fat absorption, as well as enhancing the fecal excretion of bile acids by increasing dietary fiber [43,44]. Both of these characteristics are crucial in food applications [45] as they influence the texture, nutritional value, and palatability of food products [46]. In meat products, the WHC of okara is beneficial for preserving moisture and maintaining tenderness [47], while the OHC aids in fat retention during processing, thereby contributing to flavor preservation [48]. As shown in Table 1, the WHC of the OH-treated groups significantly increased compared to the blank and control groups, with values observed for OH40-9 (0.56 g/g), OH45-6 (0.68 g/g), and OH50-3 (0.57 g/g). Among these, OH45-6 exhibited the highest WHC, representing a significant increase of 51.11% compared to the blank group. Similarly, the OHC was significantly improved across all groups, with OH45-6 (0.51 g/g) showing the highest value, corresponding to an 88.89% increase over the blank group. These enhancements may be attributed to pore formation in okara, potentially induced by the electric field and thermal effects, which resulted in a loose and porous structure that increased the surface area available for water and oil absorption, thereby facilitating the penetration and retention of water and oil molecules [49,50,51]. WHC is related to the fiber content of okara, and OH treatment may disrupt the compact structure of the fibers, leading to the exposure of more hydrophilic groups [52]. OHC is associated with the surface proteins of okara. During OH, hydrophobic amino acids on the protein surface unfold, facilitating the attachment of fats [53]. The WHC and OHC of OH40 increased with prolonged holding time, as under the influence of the electric field, the protein and fiber structures gradually unfolded with increasing holding time [54]. For OH45, the values initially increased and then decreased, while for OH50, they exhibited a continuous decline. It is noteworthy that the water-holding capacity of OH50-9 (0.39 g/g) significantly decreased. This could be attributed to excessively high field strength and prolonged holding time, which may have led to the disruption of hydrophilic groups and fiber structures in okara, resulting in decreased surface area and damaged porous structure [55,56]. Previous studies have shown that the pectin extracted from citrus peel residue after OH treatment exhibits better WHC and OHC [57], and the WHC and OHC of rapeseed have also been significantly improved [58]. In conclusion, under appropriate holding times, OH can enhance the water-holding capacity and oil-holding capacity of okara.

### 3.2. Swelling Capacity (SC)

Swelling capacity (SC) is also an indicator of the hydration capacity of okara and is closely related to its fiber–protein complex structure [59]. As shown in Table 1, OH treatment significantly increased the SC in groups OH40-9 (2.56 mL/g), OH45-6 (3.06 mL/g), OH45-9 (2.73 mL/g), OH50-3 (2.99 mL/g), and OH50-6 (2.79 mL/g). Among these, OH45-6 exhibited the highest SC, with a notable increase of 63.64% compared to the control group. This enhancement may be attributed to the loosening of fibers upon exposure to the aqueous phase after OH treatment, resulting in an increased number of exposed hydrophilic groups and the release of soluble components [60]. In contrast, treatments such as OH40-3, OH40-6, and OH45-3, which involved relatively low field strengths and short holding times, may have had limited effects on the okara structure, thereby leading to insignificant improvements in SC [61]. However, for OH50-9 (2.31 mL/g), the excessive field strength and prolonged holding time may have led to the destruction of the okara’s pore structure and surface area, thereby not significantly enhancing the SC. The experiments were well validated with each other. This trend aligned with the water-holding capacity [62]. From this, it can be inferred that under appropriate field strength and holding times, the SC of okara is significantly enhanced. The mucilage extracted from *Althaea officinalis* L. following OH treatment demonstrated an SC of 76.16% [63], and millet subjected to OH-assisted treatment also exhibited a favorable SC [64]. In conclusion, in this experiment, the WHC, OHC, and SC of the sample were more suitable at a field strength of 45 V/cm and a holding time of 6 min.

### 3.3. Total Sugar Content

**Figure 2:**
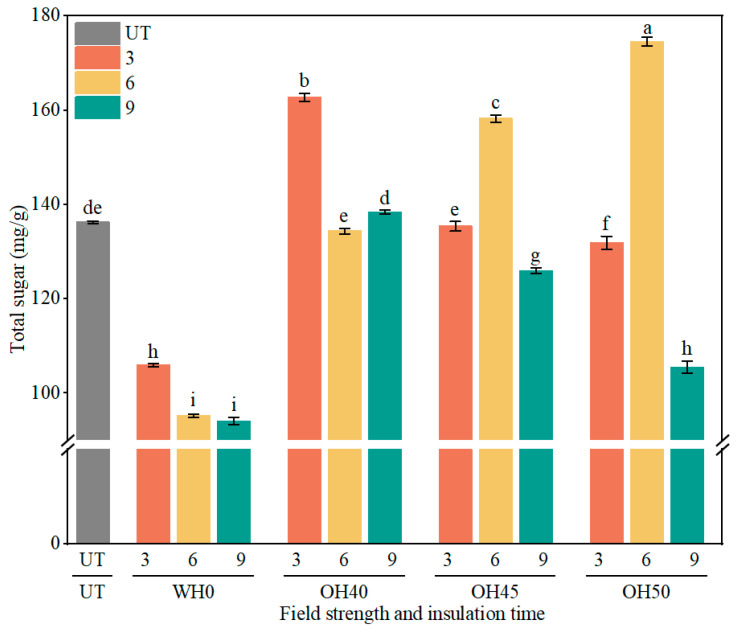
Total sugar content of okara after WH and OH treatments. UT: untreated okara, WH: water heating, OH: ohmic heating. Numbers 3, 6, and 9 represent incubation for 3, 6, and 9 min; 0, 40, 45, and 50 represent field strengths of 0, 40, 45, and 50 V/cm. The different letters represent significant differences among all groups (*p* < 0.05).

Total sugar plays a crucial role in food, affecting the taste, color, aroma, and nutritional value of the food products [65]. According to Figure 2, the total sugar content significantly decreased in all groups after WH treatment (*p* < 0.05). With the extension of incubation time, the total sugar content of the samples showed a decreasing trend, possibly due to the prolonged water bath heating time, leading to sugar degradation during the heating and incubation processes [66]. After OH treatment, the total sugar content significantly increased in certain groups, while a significant decrease was observed in others (*p* < 0.05). Among the tested groups, OH50-6 exhibited the highest total sugar content at 174.56 mg/g, followed by OH40-3 and OH45-6, with values of 162.73 mg/g and 125.89 mg/g, respectively. These results suggest that OH treatment may induce cell wall rupture and membrane disruption, thereby enhancing the release of sugar molecules [67]. Similar findings have been reported, such as the significant increase in total sugar content in watermelon rind juice following OH treatment [68], and a 74.5% increase in sugar yield from date powder after OH application [69]. However, in the OH45-9 and OH50-9 groups, the total sugar content was relatively low (*p* < 0.05), recorded at 125.89 mg/g and 105.48 mg/g, respectively. This could be attributed to prolonged incubation time leading to a reversible permeabilization effect [70], or it could be due to excessively high field strength and prolonged incubation time causing damage to sugar molecules, resulting in a decrease in total sugar content [71]. Therefore, appropriate OH treatment can effectively increase the total sugar content in soy pulp, thereby facilitating the release and utilization of sugars.

### 3.4. Bioactive Compounds

#### 3.4.1. SDF

**Figure 3:**
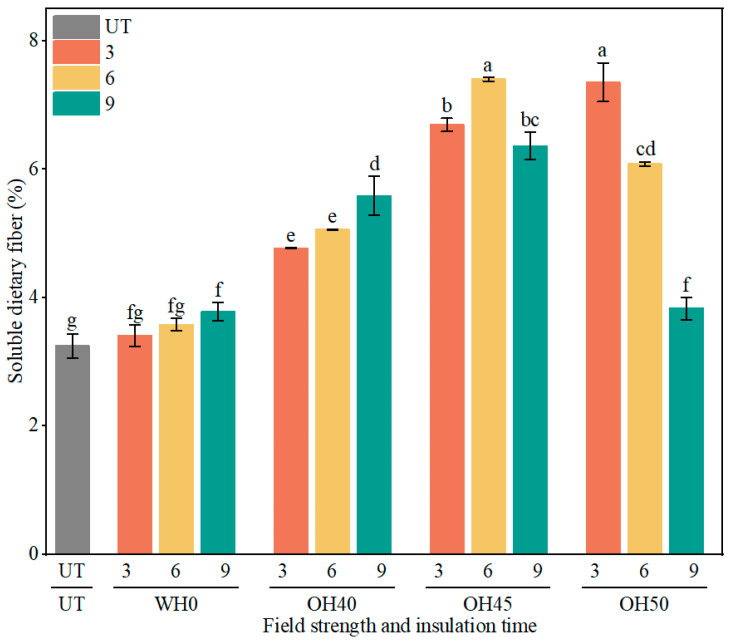
Content of SDF in okara after WH and OH treatment. UT: untreated okara, WH: water heating, OH: ohmic heating. Numbers 3, 6, and 9 represent incubation for 3, 6, and 9 min; 0, 40, 45, and 50 represent field strengths of 0, 40, 45, and 50 V/cm. The different letters represent significant differences among all groups (*p* < 0.05).

SDF is an important prebiotic that plays a crucial role in reducing plasma cholesterol, enhancing immune regulatory activity, and exhibiting excellent gelation and emulsification abilities [72]. As shown in Figure 3, compared to the blank group, the content of SDF in the samples gradually increased with the prolongation of WH holding time. When insulated for 9 min, there was a significant increase in the SDF content in the samples (*p* < 0.05). The degradation or swelling of the cellulose and hemicellulose fractions, potentially induced by heat treatment, may lead to their conversion into soluble dietary fiber, thereby causing a gradual increase in soluble dietary fiber content [73]. OH treatment significantly enhanced the soluble dietary fiber content in the samples, with the highest level observed in OH45-6 (7.40%), followed by OH50-3 (7.36%) and OH45-3 (6.69%). The OH process likely facilitated the breakdown of long insoluble fibers into shorter fragments, improving their digestibility and solubility [74]. Simultaneously, during OH treatment, the electric field induced both the thermal and non-thermal degradation of cell structures, leading to the disruption of the food cell walls and the release of soluble dietary fibers [75]. After the OH treatment of corn flour, the SDF content in the samples increased by 65% [31]. When applied to grape pomace, OH resulted in a 25% increase in soluble dietary fiber content in the samples [76]. At low field strengths (40 V/cm), the SDF content gradually increased with prolonged holding time. However, at higher voltages (45 V/cm and 50 V/cm), the SDF content in the samples showed an initial increase followed by a decrease and a linear decline with increasing holding time. When the holding time was short (3 min), the SDF content in the samples gradually increased with the increase in field strength. However, with longer holding times (6 min and 9 min), as the field strength increased, the SDF content exhibited a trend of initially rising and then declining. Particularly in the case of OH50-9, there was a significant decrease in the SDF content of the sample (*p* < 0.05). The content of SDF in okara is influenced by both the holding time and the electric field strength. An excessive voltage or prolonged holding time can lead to the loss and degradation of soluble dietary fiber instead [77].

#### 3.4.2. TFC

**Figure 4:**
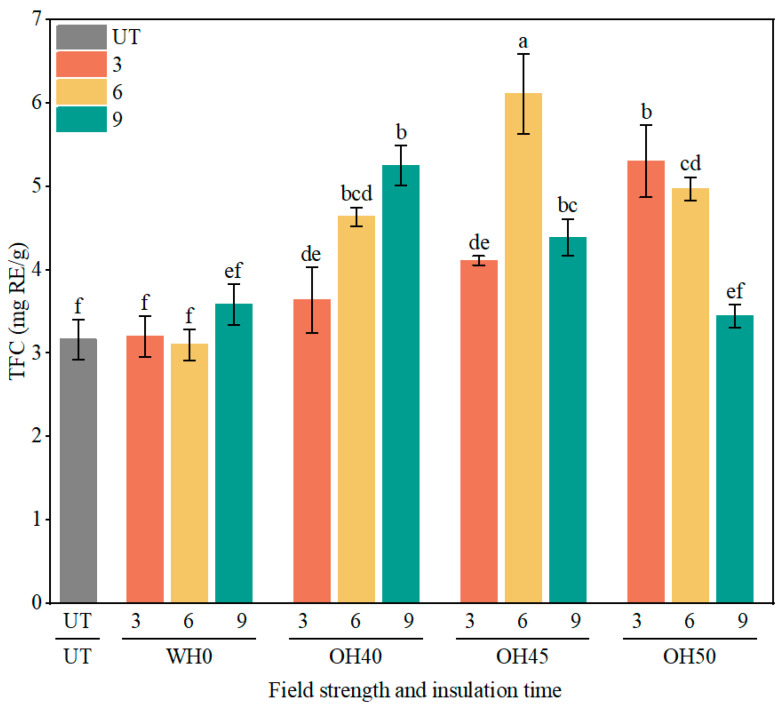
The TFC of okara before and after WH and OH treatments. TFC: total flavonoid content, UT: untreated okara, WH: water heating, OH: ohmic heating. The numbers 3, 6, and 9 represent incubation for 3, 6, and 9 min; 0, 40, 45, and 50 represent field strengths of 0, 40, 45, and 50 V/cm. The different letters represent significant differences among all groups (*p* < 0.05).

TFC represents the measurement of flavonoid substances [78]. These compounds are beneficial to human health, demonstrating activities such as anti-diabetic, antioxidant, and anticancer effects [79]. As illustrated in Figure 4, no significant change in total flavonoid content was observed following water heating (WH) treatment. In contrast, the total flavonoid content exhibited significant variation after ohmic heating (OH) treatment (*p* < 0.05). The highest flavonoid content was detected in OH45-6 at 6.11 mg RE/g, followed by OH50-3 at 5.31 mg RE/g and OH40-9 at 5.25 mg RE/g. This increase is attributed to the electroporation effect induced by OH treatment, which permeabilizes the cell walls, leading to their disruption and facilitating the release of phenolic acids, flavonoids, and other phytochemicals [80]. According to existing reports, OH treatment can effectively increase the TFC in food. Compared to traditional heating methods, the TPC in cornflakes increased by at least 1.63 times after OH treatment [81]. After OH treatment, the TFC in mango juice was significantly higher compared to traditional heating methods [82]. When the holding time was 3 min, with an increase in field strength, flavonoid substances dissociate effectively. However, when the holding time was 6 min and 9 min, the TFC showed an initial increase followed by a decrease with increasing field strength. This could be attributed to the degradation of flavonoid components with the increase in field strength and, leading to a decline in TFC. It is worth noting that when the holding time was 9 min, the TFC of OH50 (3.45 mg RE/g) was significantly lower than the other groups (*p* < 0.05), which could have been caused by thermal degradation possibly induced by the longer holding time [83]. Therefore, the OH treatment of okara can effectively increase the TFC, influencing its nutritional value and functionality, thereby enhancing its application value in functional foods.

#### 3.4.3. TPC

**Figure 5:**
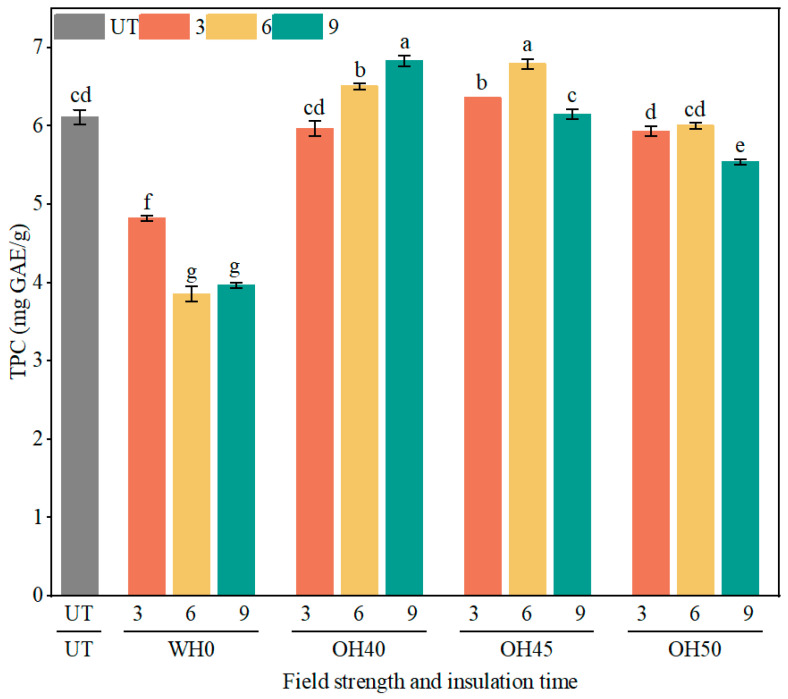
The TPC of okara after WH and OH treatments. TPC: total phenolic content, UT: untreated okara, WH: water heating, OH: ohmic heating. The numbers 3, 6, and 9 represent incubation for 3, 6, and 9 min; 0, 40, 45, and 50 represent field strengths of 0, 40, 45, and 50 V/cm. The different letters represent significant differences among all groups (*p* < 0.05).

Phenolic compounds have many health benefits for the human body, as they possess high antioxidant properties that can improve human health, such as anti-diabetic and anti-cardiovascular disease effects [84]. As shown in Figure 5, the TPC of all groups significantly decreased after WH treatment (*p* < 0.05), and decreased with increasing incubation time. Phenolic compounds had poor stability and were prone to degradation and loss during heating [85], leading to a decrease in their content. Additionally, incubation time also affected their structure and content [86]. After OH treatment, the TPC of some samples significantly increased (*p* < 0.05). Among them, the TPC of OH40-9 was the highest, at 6.83 mg GAE/g, followed by OH45-6 and OH40-6, which were 6.80 mg GAE/g and 6.51 mg GAE/g, respectively. The OH treatment duration was shorter than the WH treatment, thereby reducing heat damage to phenolic compounds [87]. Simultaneously, the OH process membrane permeabilization, making bioactive substances more easily extractable, leading to an increase in the TPC of the samples [88]. It is noteworthy that the TPC of the OH50 treatment group exhibited a decreasing trend, with OH50-9 showing a significantly lower TPC compared to the other OH treatment groups (*p* < 0.05). This decline may be attributed to the excessively high field strength and rapid heating, which likely caused a loosening of the sample’s intercellular structure [89], leading to the degradation of active components. Furthermore, prolonged holding time also contributed to the reduction in TPC [90]. The TPC extraction rate of pineapple cores were improved after OH treatment [91], and the TPC of grapefruit juice increased by 10% following OH sterilization [92]. Therefore, under appropriate field strength and suitable holding time, OH treatment can effectively increase the TPC in okara. In this experiment, conditions such as OH40-9 and OH45-6 were found to be more suitable.

### 3.5. Antioxidant Capacity Analysis (DPPH and ABTS)

**Figure 6:**
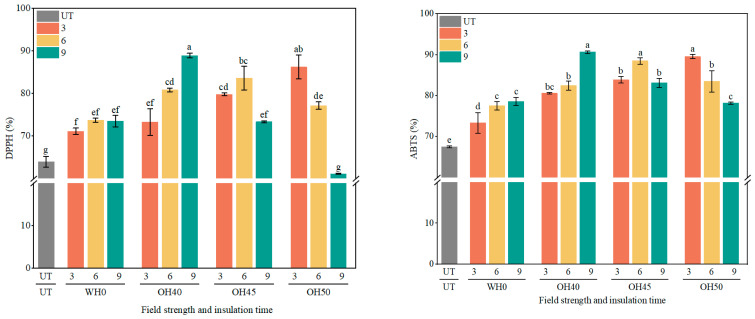
The DPPH radical scavenging and ABTS radical scavenging abilities of okara after WH and OH treatments. UT: untreated okara, WH: water heating, OH: ohmic heating. The numbers 3, 6, and 9 represent incubation for 3, 6, and 9 min; 0, 40, 45, and 50 represent field strengths of 0, 40, 45, and 50 V/cm. The different letters represent significant differences among all groups (*p* < 0.05).

In Figure 6, the DPPH and ABTS radical scavenging capacities of okara after WH and OH treatments are presented. It was observed that the antioxidant capacity of the samples increased after both WH and OH treatments (*p* < 0.05). Studies suggested that heat treatment promoted the generation of pro-oxidants, antioxidants, and new antioxidant components [93]. For the DPPH radical scavenging capacity of okara, except for OH50-9, the DPPH of all other groups showed a significant increase. Among the samples, OH40-9 exhibited the highest DPPH radical scavenging activity (88.95%), followed by OH50-3 (86.24%) and OH45-6 (83.64%). These values represent significant increases of 39.07%, 34.83%, and 30.76%, respectively, compared to untreated okara (63.96%) (*p* < 0.05). This enhancement may be attributed to the electric field promoting the hydrolysis of glycosidic bonds in phenolic compounds, thereby increasing the antioxidant activity of the extract [94]. Regarding the ABTS radical scavenging capacity, both water heating (WH) and ohmic heating (OH) treatments significantly improved the antioxidant ability of the samples. Among them, OH40-9 showed the highest ABTS scavenging activity (90.60%), followed by OH50-3 (89.54%) and OH45-6 (88.49%). These represented significant increases of 34.22%, 32.65%, and 31.10%, respectively, compared to the untreated control (67.50%) (*p* < 0.05). Through observation, it was found that the antioxidant capacity increased with prolonged incubation time when no field strength was applied and when the field strength was 40 V/cm. When the field strength was 45 V/cm, the antioxidant capacity initially increased and then decreased with prolonged incubation time. At higher field strengths, the antioxidant capacity decreased with increasing incubation time, possibly due to the degradation and oxidation of phenolic compounds caused by excessively high field strength and prolonged incubation time [91]. At an incubation time of 3 min, both DPPH and ABTS radical scavenging capacities exhibited an increasing trend with rising field strength. However, for incubation times of 6 and 9 min, these antioxidant activities initially increased and subsequently decreased as the field strength increased, indicating that antioxidant capacity is influenced by the applied field strength. Previous studies have demonstrated that field strength significantly affects cell wall permeabilization rate, the release of intracellular molecules, diffusion rates, and the activity of bioactive compounds [95]. After OH treatment, compounds such as isoflavones [78], tannic acid [91], lignin, and hydroxycinnamic acid [96] in okara are affected, thereby influencing the antioxidant capacity of okara. The extracts of olive leaves after OH treatment exhibited enhanced antioxidant capacity [95]. After OH treatment, the antioxidant ability of the fruit peel was significantly improved [97].

Therefore, the antioxidant capacity of okara is influenced by the field strength and incubation time. When the incubation time and field strength are appropriate, the antioxidant capacity of okara can be significantly enhanced. Overall, in this experiment, the antioxidant capacity of OH40-9 was found to be relatively favorable. Simultaneously, the antioxidant capacity test results were related to the increase in total phenolic and total flavonoid content, providing good validation between the experiments.

### 3.6. Fourier-Transform Infrared Spectroscopy (FTIR)

**Figure 7:**
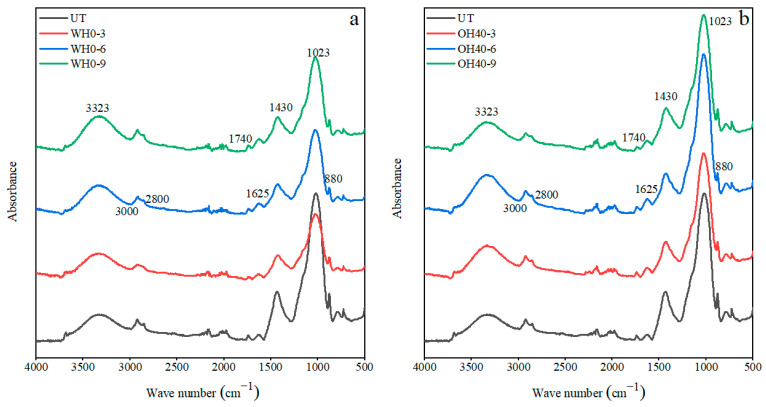

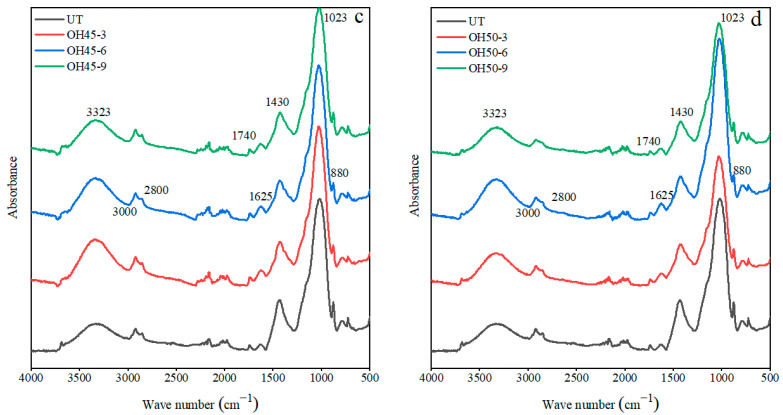
The Fourier-transform infrared spectra of okara after WH and OH treatment. UT: untreated okara, WH: water heating, OH: ohmic heating. The numbers 3, 6, and 9 represent incubation for 3, 6, and 9 min; 0, 40, 45, and 50 represent field strengths of 0, 40, 45, and 50 V/cm. (**a**) Fourier-transform infrared spectra of UT and WT. (**b**) Fourier-transform infrared spectra of UT and OH40. (**c**) Fourier-transform infrared spectra of UT and OH45. (**d**) Fourier-transform infrared spectra of UT and OH50.

Figure 7 displays the FTIR spectrum of the sample, revealing peaks associated with polysaccharides, proteins, and lipids [98,99]. The strong and broad absorption peak at 3323 cm^−1^ corresponded to the stretching vibration of hydroxyl groups (-OH) [100], indicating the presence of sugars such as arabinose and xylose, as well as phenolic substances [101]. After WH, the absorption peaks at this region all intensified, possibly indicating the liberation of -OH groups from polysaccharides under high-temperature incubation [102]. However, compared to the WH treatment, the enhancement of absorption peaks in the OH-treated group was more pronounced. The absorption peak at 1430 cm^−1^ corresponded to the vibrations of the -OH or -CO groups in hemicellulose [103], while the peak at 1023 cm^−1^ was attributed to the C–O stretching vibration, originating from the C–O–H and C–O–C groups of the sugar rings in cellulose and hemicellulose [104]. Following water treatment (WH), absorption peaks in this region were weakened across all groups; however, in the OH-treated samples, these absorption peaks either remained unchanged or were enhanced. This suggests that OH treatment may enhance the absorption of polysaccharides, possibly due to accelerated intermolecular motion induced by the electric field, facilitating more effective liberation of polysaccharide functional groups [105]. The small, sharp peak observed at 880 cm^−1^ is characteristic of the β-glycosidic bond present in hemicellulose [106]. The absorption peak at 1625 cm^−1^ corresponds to the amide I band of proteins [107]. Post OH treatment, this peak was intensified, indicating that OH treatment can influence the secondary structure of proteins, consistent with findings reported by Wang et al. [108] and Avelar et al. [109]. The spectral region between 3000 cm^−1^ and 2800 cm^−1^, along with the absorption peak at 1740 cm^−1^, are associated with lipids [110]. Notably, in all OH50 groups, a marked attenuation of absorption peaks between 3000 cm^−1^ and 2800 cm^−1^ was observed, likely due to the high field strength. Furthermore, with increasing incubation time, the absorption peaks progressively diminished. It can be inferred that OH treatment has an impact on the secondary structure of okara, which is consistent with existing research [63,111]. It affects polysaccharides, proteins, and lipids, particularly the polysaccharide structure.

### 3.7. Microstructure

**Figure 8:**
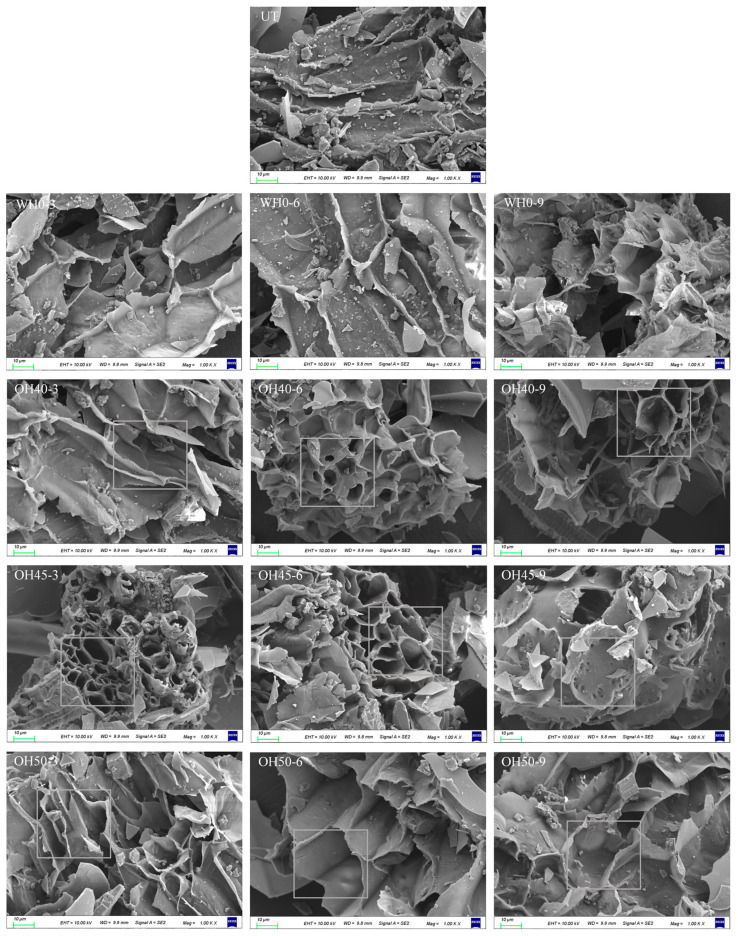
The microstructure of okara after WH and OH treatment. UT: untreated okara, WH: water heating, OH: ohmic heating. The numbers 3, 6, and 9 represent incubation for 3, 6, and 9 min; 0, 40, 45, and 50 represent field strength of 0, 40, 45, and 50 V/cm. The square in images emphasizes the pores of okara.

As indicated by Figure 8, OH resulted in changes to the tissue structure of okara, influenced by varying field strengths and holding times. The surface of untreated okara appears relatively flat, but upon heating to 100 °C, the sample surface wrinkles, with this effect becoming more pronounced with increasing holding time. In the OH-treated group, the pore structure of okara increased, leading to an enlarged surface area, which may enhance the sample’s adsorption functionality [112]. This finding aligns with previous research on OH treatment improving WHC and OHC. In OH40, when incubated for 3 min, the surface of the sample becomes more wrinkled. After 6 min of incubation, pores begin to form, and by 9 min of incubation, the pores become pronounced, leading to an increase in porosity and surface area, consequently enhancing the adsorption capacity [113]. In the OH45 and OH50 groups, increasing the holding time led to the gradual enlargement and disordering of the sample pores, resulting in a looser microstructure. This phenomenon may be attributed to the rapid heating induced by higher electric field intensities and extended holding times, which potentially caused structural damage to the samples [114]. Notably, in the OH45-9 group, a finer pore structure was observed on the sample surface, possibly due to electroporation effects induced by excessively high field strength and prolonged exposure [115]. However, it should be noted that the samples prepared for scanning electron microscopy were subjected to vacuum freeze-drying, which may have altered their original structure [116]. Therefore, OH treatment can influence the structural integrity of okara, thereby affecting its adsorption properties.

### 3.8. Correlation Analysis

**Figure 9:**
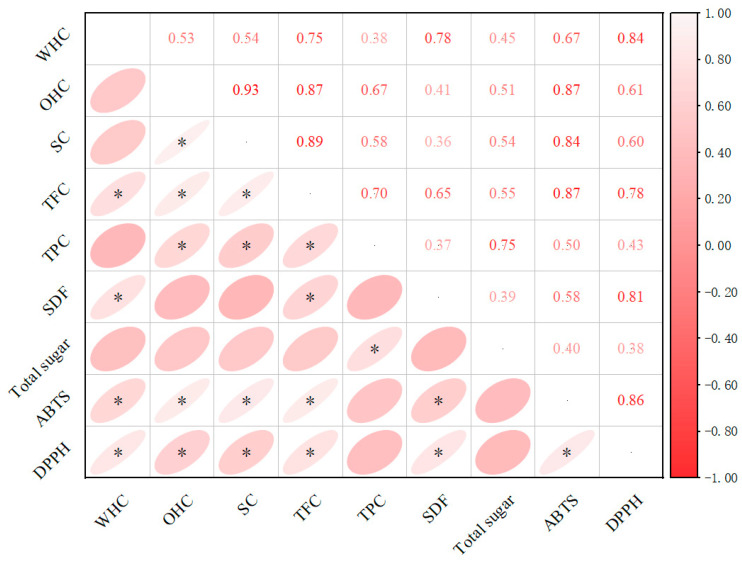
Correlation analysis among various parameters of okara after ohmic heating modification. WHC: water-holding capacity, OHC: oil-holding capacity, SC: swelling capacity, TFC: total flavonoid content, TPC: total phenolic content, SDF: soluble dietary fiber. ‘*’ indicates a significant difference (*p* < 0.05).

Pearson correlation analysis was used to explore the relationships between the physical properties, bioactive compounds, and antioxidant capacity of okara after OH modification (Figure 9). The results showed that, after modification, WHC was significantly correlated with TFC, SDF, and antioxidant capacity (*p* < 0.05), which may be attributed to the OH-induced release and structural reorganization of flavonoids, soluble dietary fiber, and antioxidant compounds. OHC was significantly correlated with SC, TFC, TPC, and antioxidant capacity (*p* < 0.05), likely due to the disruption of the cell wall structure by OH, which increased the porosity of okara and promoted the enrichment of phenolic compounds, thereby enhancing its oil-binding capacity. SC was significantly correlated with TFC, TPC, and antioxidant capacity (*p* < 0.05), which could be attributed to the release and redistribution of flavonoids, phenolics, and antioxidant compounds induced by OH, leading to an improved fiber network structure and enhanced swelling capacity. SDF was significantly correlated with TFC (*p* < 0.05), possibly due to the OH-induced disruption of the cell wall binding structures, which promoted the simultaneous release of bound flavonoids and soluble dietary fiber. Total sugar was significantly correlated with TPC (*p* < 0.05), as OH accelerated the co-release of cell wall polysaccharides and bound phenolic compounds.

Overall, OH significantly influenced the WHC, OHC, SC, and related functional properties of okara by disrupting the cell wall structure and promoting the release and reorganization of bioactive components such as flavonoids, phenolics, polysaccharides, and dietary fiber, thereby resulting in significant correlations among various physicochemical parameters.

## 4. Conclusions

The research found that OH treatment improved some properties of okara. The results indicated that ohmic heating significantly enhanced the physical properties of okara, with the water-holding capacity (WHC), oil-holding capacity (OHC), and swelling capacity (SC) increasing by 51.11%, 88.89%, and 43.64%, respectively. Additionally, ohmic heating positively affected the microstructure and secondary structure of okara, thereby enhancing its functional characteristics. Ohmic heating also led to a 7.40% increase in soluble dietary fiber (SDF) content, with total sugar content reaching 174.56 mg/g, significantly improving its antioxidant properties and offering greater potential for the development of prebiotic components.

The experimental results further demonstrated that the electric field strength and insulation time are key factors influencing the properties of okara. High-quality okara could only be obtained under appropriate electric field strengths and insulation conditions. In this experiment, considering the various properties of modified okara, the most suitable conditions were found to be an electric field strength of 45 V/cm and an insulation time of 6 min. Ohmic heating not only improved the nutritional value and functionality of okara but also provided broad prospects for its application in the food industry. By enhancing the physical and chemical properties of okara, ohmic heating opens up new possibilities for its use in functional foods, nutritional supplements, and sustainable food processing. This approach holds promise in terms of adding higher value to the food industry, promoting the development and production of green and healthy foods.

## Figures and Tables

**Table 1:** WHC, OHC, and SC of okara.

Code	WHC (g/g)	OHC (g/g)	SC (mL/g)
UT	0.45 ± 0.01 ^g^	0.27 ± 0.01 ^g^	1.87 ± 0.07 ^f^
WH0-3	0.49 ± 0.01 ^e,f^	0.31 ± 0.01 ^f,g^	2.07 ± 0.01 ^e,f^
WH0-6	0.51 ± 0.01 ^d,e,f^	0.33 ± 0.01 ^f^	2.04 ± 0.12 ^e,f^
WH0-9	0.51 ± 0.01 ^c,d,e^	0.31 ± 0.01 ^f,g^	2.09 ± 0.05 ^e,f^
OH40-3	0.52 ± 0.01 ^c,d,e^	0.4 ± 0.01 ^e^	2.33 ± 0.17 ^d,e^
OH40-6	0.54 ± 0.01 ^b,c,d^	0.42 ± 0.01 ^c,d,e^	2.39 ± 0.19 ^c,d,e^
OH40-9	0.56 ± 0.01 ^b^	0.43 ± 0.01 ^c,d,e^	2.56 ± 0.08 ^b,c,d^
OH45-3	0.54 ± 0.01 ^b,c^	0.45 ± 0.02 ^c,d^	2.39 ± 0.23 ^c,d,e^
OH45-6	0.68 ± 0.01 ^a^	0.51 ± 0.01 ^a^	3.06 ± 0.06 ^a^
OH45-9	0.48 ± 0.01 ^f,g^	0.46 ± 0.01 ^b,c^	2.73 ± 0.17 ^a,b,c,d^
OH50-3	0.57 ± 0.02 ^b^	0.49 ± 0.01 ^a,b^	2.99 ± 0.11 ^a,b^
OH50-6	0.51 ± 0.01 ^c,d,e,f^	0.42 ± 0.02 ^d,e^	2.79 ± 0.23 ^a,b,c^
OH50-9	0.39 ± 0.01 ^h^	0.42 ± 0.01 ^d,e^	2.46 ± 0.24 ^c,d,e^

WHC: water-holding capacity, OHC: oil-holding capacity, SC: swelling capacity, UT: untreated okara, WH: water heating, OH: ohmic heating. Numbers 3, 6, and 9 represent incubation for 3, 6, and 9 min; 0, 40, 45, and 50 represent field strengths of 0, 40, 45, and 50 V/cm. Different letters indicate significant differences (*p* < 0.05) within the same column.

## Data Availability

The original contributions presented in the study are included in the article, further inquiries can be directed to the corresponding author.
